# Acquired Hemophilia A in Peripartum Period: Diagnostic and Therapeutic Dilemma

**DOI:** 10.7759/cureus.16803

**Published:** 2021-08-01

**Authors:** Syed Hamza Bin Waqar, Leen Khoury, Ather Hussain, Isabel M McFarlane

**Affiliations:** 1 Hematology and Oncology, SUNY Downstate Medical Center, New York, USA; 2 Hematology and Oncology, Rochester General Hospital, Rochester, USA; 3 Internal Medicine, SUNY Downstate Medical Center, New York, USA

**Keywords:** acquired hemophilia a, factor viii, hemophilia, aptt, peripartum, aha

## Abstract

Acquired hemophilia A (AHA) is a bleeding diathesis caused by auto-antibody generation against factor VIII, an essential component of the coagulation cascade. Although having many etiologies, pregnancy is also one of the conditions associated with AHA. It mostly presents as a raised activated partial thromboplastin time (aPTT), and during the peripartum and postpartum period, concern for AHA should be raised as delays in diagnosis can be detrimental. Herein, we present a case of a 31-year-old female with sickle cell trait who developed venous bleeding and, later, neuraxial, musculoskeletal, and subcutaneous bleeding. She underwent an extensive course of treatment before getting into remission.

## Introduction

Factor VIII is an essential factor in the coagulation cascade pathway's intrinsic component due to its procoagulant functionality. Sometimes, owing to insults that could be autoimmune or for reasons not fully known, the immune system in particular B cells generates a response against the complex of factor VIII leading to acquired hemophilia A, which can present in various ways, ranging anywhere from being asymptomatic to life-threatening bleeds. Given the scarcity of this condition, especially in cases occurring in pregnancy, the literature does not have sufficient evidence or controlled trials to manage bleeding and immunosuppression [1-4]. We present a unique case of peripartum hemophilia A, which appeared in a young African American female with sickle cell trait with unusual bleeding sites who needed an extensive therapy course to achieve hemostasis.

## Case presentation

A 31-year-old woman with sickle cell trait, para 1+1, who delivered a healthy baby via cesarean section developed injection-site hematoma over the triceps and the site of the incision. She also had an episode of profuse bleeding from intravenous lines placed. She was transfused with four fresh frozen plasma (FFP) units over a few hours, along with tranexamic acid and misoprostol. After receiving these products in the labor and delivery unit, the bleeding did not stop. The patient further noticed moderate back pain with progressive weakness in her lower extremities bilaterally with some numbness and tingling sensation over the dorsum and lateral aspect with no saddle anesthesia, bowel, or bladder incontinence. The bleeding did not stop after getting hemostatic agents and FFPs. The patient became hypotensive and was resuscitated with fluids. The physical exam was unremarkable. Labs were sent, which was significant for a drop of hemoglobin of 4.5 g/dl from her baseline with a prolonged partial thromboplastin time (PTT) of 83 seconds, markedly raised from preoperative PTT of 49 seconds, which was not followed up right away, given an emergent cesarean section need of the patient.

**Figure 1:**
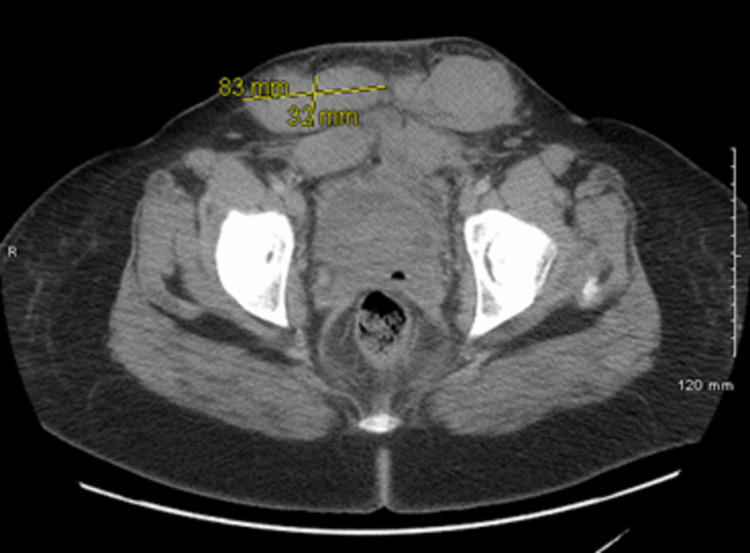
Computed tomography axial view showing hematoma of 8.3 cm x 3.2 cm in subcutaneous soft tissue

**Figure 2:**
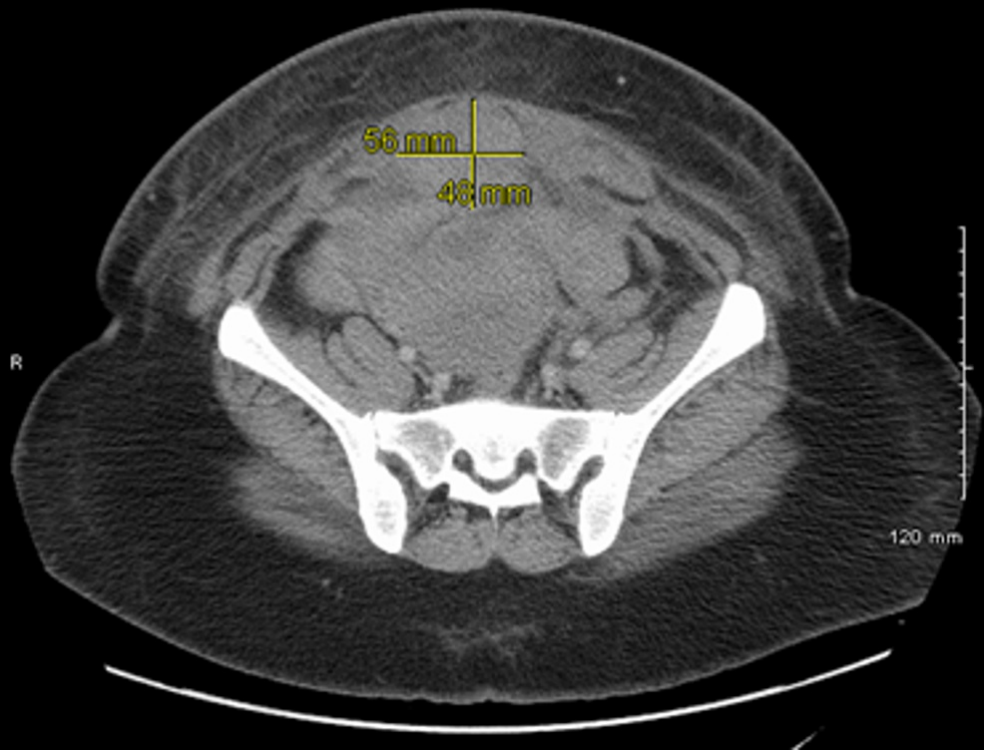
Computed tomography axial view showing the hematoma anterior to the surgical line of the uterus (5.6 cm x 4.8 cm)

The patient was then started on activated prothrombin complex concentrate (aPCC), also known as FEIBA (factor eight inhibitor bypassing activity) at 50 units/kg (U/kg). Labs were sent to determine the causation of bleeding. Given the emergent concern for retroperitoneal and epidural bleeding and concerning spinal cord compression symptoms, we obtained a computed tomographic (CT) scan of the abdomen and pelvis and magnetic resonance (MRI) of the thoracic and lumbar spine. CT abdomen pelvis revealed a hematoma anterior to the uterus's surgical line with 5.6 cm x 4.8 cm x 6.2 cm (anteroposterior [AP] x transverse x craniocaudal) dimensions along with two other hematomas within the subcutaneous soft tissues measuring 8.3 cm x 3.2 cm x 4.0 cm and 7.2 cm x 4.9 cm x 6.2 cm with enlargement of the rectus muscles bilaterally representing intramuscular hematoma (Figures 1, 2).

**Figure 3:**
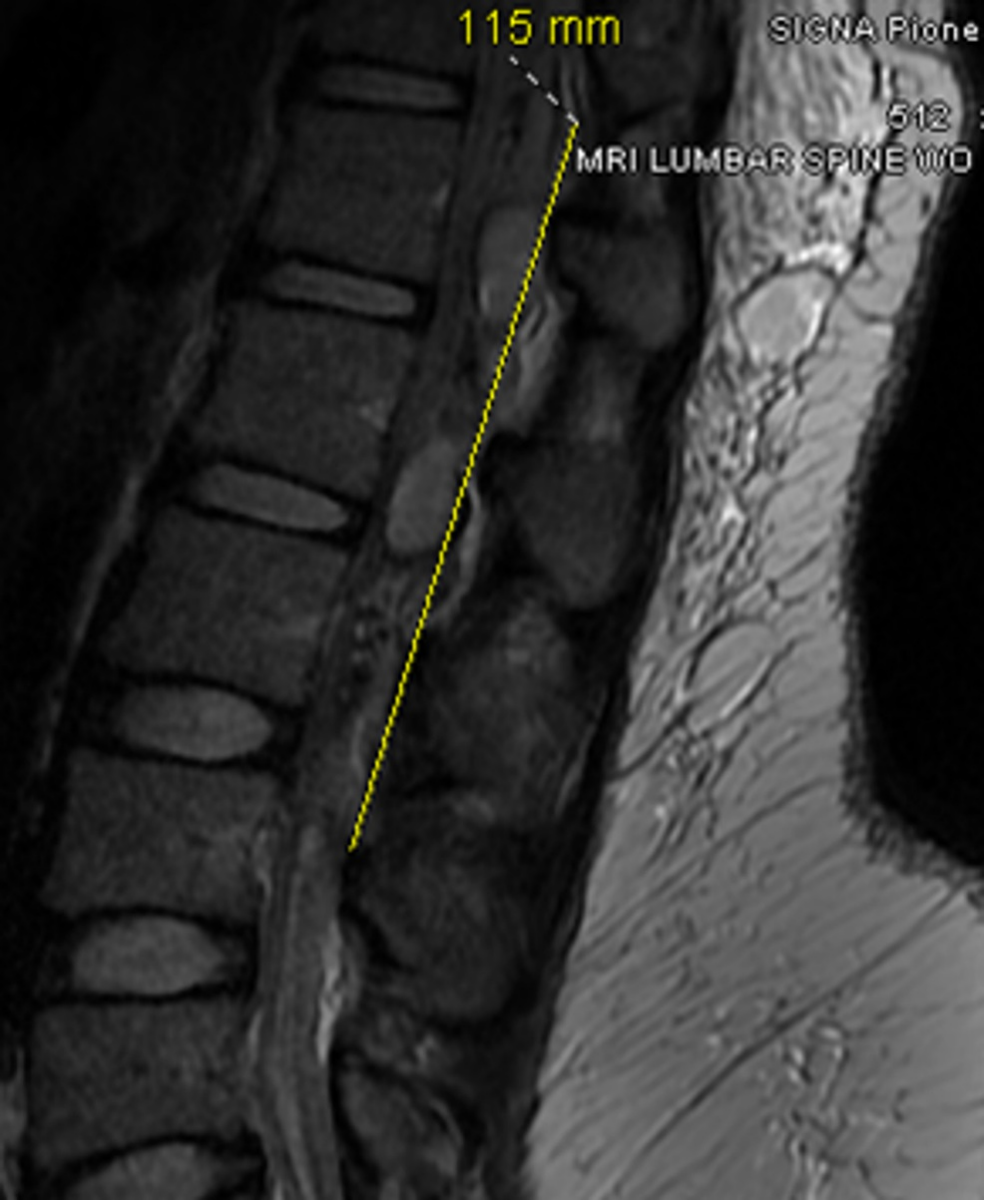
Sagittal T2 MRI of the lumbar spine without contrast showing the craniocaudal extension of the hematoma of 11.5 cm extending from T12/L1 to L4 level MRI, Magnetic resonance imaging.

**Figure 4:**
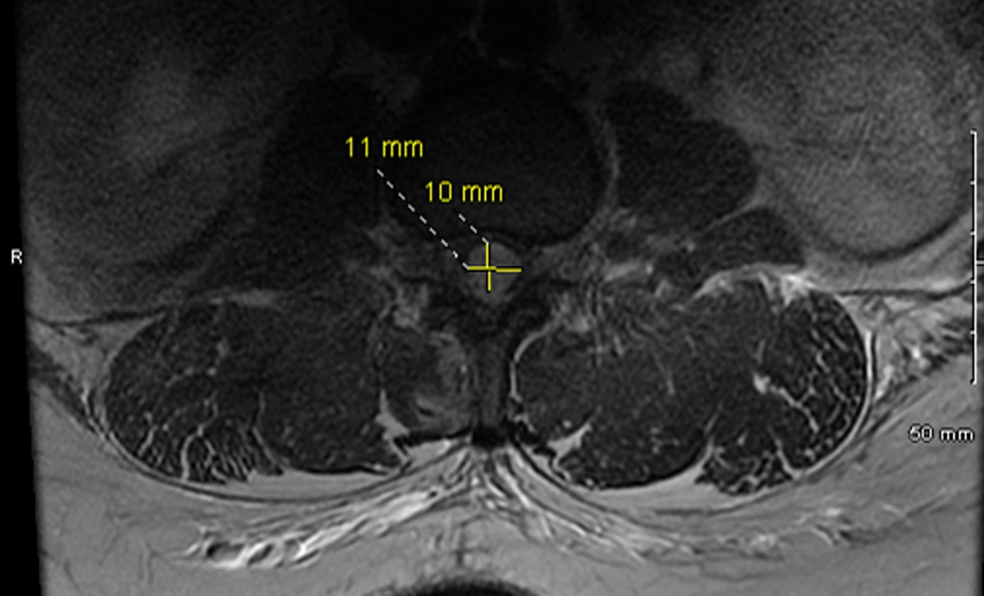
T2 axial MRI of the lumbar spine without contrast showing the transverse view of the hematoma measuring 1.1 cm x 1 cm MRI, Magnetic resonance imaging.

A heterogeneous signal within the spinal canal's posterior epidural space consistent with an epidural hematoma was seen on the MRI lumbar spine. It extended from the T12/L1 level downward to approximately the L4 level, measured 1.0 cm AP x 1.1 cm transverse and at least 11.5 cm craniocaudal. A significant mass effect on the cauda equina and conus medullaris was observed (Figures 3, 4).

The hemoglobin was further dropped on the second postoperative day. Given the continued bleeding risk, the patient was given recombinant factor VIIa at 90 microgram/kg two times along with another infusion of aPCC (at 50 U/kg). Three units of red blood cell transfusions were given concomitantly.

Lab work sent earlier came back with a factor VIII level of below 2% with inhibitor levels of 72 Bethesda units (BU). Fibrinogen, lactate dehydrogenase (LDH), uric acid, antiphospholipid antibodies along with diluted Russell’s viper venom time (DRVVT), and silica clotting time came back normal as part of the work-up excluding lupus anticoagulant and disseminated intravascular coagulation. Diagnosis of acquired hemophilia A (factor VIII) was made. Treatment was directed to control and prevent bleeding and to eradicate the inhibitors.

Her symptoms improved, and hemoglobin, along with PTT, was trended (Table 1). The patient was discharged home on aminocaproic acid 10 days after delivery. However, she came back to the emergency department as she noted oozing of blood from her Pfannenstiel incision two days later. The decision was made to start rituximab at 375 mg/m^2^ weekly for four cycles and prednisone at 1 mg/kg and weekly monitoring of PTT and monthly inhibitor assay (Table 1).

**Table 1:** Selected laboratory data obtained during the hospital course CYC, Cyclophosphamide.

Postoperative Day (POD)	Factor VIII (%), Normal: >50%	Factor VIII Inhibitor in BU (Bethesda Units), Normal: Undetectable	Activated Prothrombin Time (aPTT), Normal: 25-37 seconds
POD 1	<2	72	83
POD 10 (after bridging and hemostatic therapy)	3	84	>120
POD 14 (initiation of rituximab and prednisone)	<2	78.7	98
POD 42 (completion of rituximab and prednisone)	4	96	104
POD 56 (after CYC and prednisone)	76	2	33
POD 84 (surveillance week 2)	87	Undetectable	25

Despite completing the rituximab and prednisone course, she continued to have elevated inhibitor levels of 96 BU and factor VIII levels at around 1%-4%; thus, prednisone was continued. Cyclophosphamide at 1.5 mg/kg was further added, and on further follow-up in two weeks, the inhibitor level came down to 2 BU and factor VIII of 76%. Prednisone was later tapered, and the patient was followed up in the hematology clinic with later titers undetectable for factor VIII inhibitors and normalization of PTT and factor VIII.

## Discussion

Autoantibodies causing coagulation factor deficiency are rare, although known for a long time. Among the coagulation factors, factor VIII is the most targeted factor that may manifest in various ways. It can range anywhere from minor bleeding to life-threatening blood loss. It is termed acquired hemophilia A. Incidence of such autoantibodies is 1.4 per million people per year [1]. Approximately, 40%-50% of cases have underlying conditions, including autoimmune disorders, malignancy, medication history, or pregnancy. Pregnancy contributes about 7%-11% of disease burden with mechanisms unclearly understood [2-4].

Biologically, factor VIII needs to become proteolytically activated to work as a cofactor for factor IXA during intrinsic pathway factor X activation by interacting with phospholipids over the platelet surface membrane. Factor VIII's procoagulant action is decreased by antibodies interacting and inhibiting the aforementioned processes [5].

Factor VIII is a soluble monovalent and cannot generate or stimulate B-cell receptor response on its own. The B-cell population is incredibly unique in acquired hemophilia A as these cells respond to blood-borne antigens that lack pathogen-associated molecular patterns (PAMPs). Theoretically, for B cells to generate antibodies against soluble monovalent such as factor VIII itself, factor VIII needs to combine higher molecular weight species, leading to subsequent activation of the B-cell population [6,7]. Polyclonal IgG has been noted to be the predominant antibody in AHA with an increased subtype IgG4, which comprises about 5% of the IgG population subset. Since IgG4 does not fix complement, AHA does not appear to be an immune complex disease [8].

Clinically, AHA cases in pregnancy mostly appear incidentally with an unexpected high aPTT level, suggesting a problem in the intrinsic pathway mentioned earlier [9]. Most data to date have been extracted from European Acquired Hemophilia (EACH2) Registry. The most common sites noted were primarily subcutaneous, mucosal, musculoskeletal, and retroperitoneal [2]. A few cases have been documented in literature mentioning the involvement of the pleura and central nervous system in AHA [10]. For reasons not understood, unlike congenital hemophilia, AHA rarely causes hemarthrosis. The appearance of bleeding also widely varies and can range anywhere from delivery to six weeks [2,11].

Raised aPTT in pregnancy must raise suspicion of AHA and result in a reflex mixing study test that will not correct and suggest the presence of autoantibodies against factor VIII. After identification of the inhibitor as autoantibodies against factor VIII, the titers are determined using Bethesda assay [11]. The patient's plasma is mainly preincubated with normal plasma for two hours at 37°C; the plasma inhibitors take minutes to hours to maximally inhibit factor VIII. Inhibitor titer is thus defined as the dilution of the patient plasma that produces 50% inhibition of factor VIII activity and is expressed as Bethesda units per millimeter (BU/ml). Informal classification helps stratify AHA with titers below 5 BU/ml considered as low and above 5-10 BU/ml as high. Bethesda assay may further be adjusted with Nijmegen modification, which decreases the false-positive low-titer inhibitors [11-13]. Inhibitors in the case of AHA are also classified into two main variants depending on their kinetics and the extent of inactivation:

1. Type 1 inhibitors follow second-order kinetics and inactivate factor VIII completely.

2. Type 2 inhibitors exhibit complex kinetics and do not inactivate factor VIII completely.

The distinction between the two types, however, has no role in clinical practice [14]. AHA can further be stratified based on factor VIII (fVIII) levels, although it should not be used to judge the severity of bleeding:

1. Factor VIII of <1%: severe

2. Factor VIII of 1%-5%: moderate

3. Factor VIII of 5% or above but less than 40% of normal: mild [15].

Our case presented severe AHA with a rare complication of neuro-axial bleed, and a mixed picture of musculoskeletal and subcutaneous bleed occurring at the day of delivery is rare, given most cases appear later in the postpartum period.

Multiple therapeutic regimens have been considered and utilized for the treatment of AHA. Replacement of factor VIII and initiating desmopressin can work in very low titers but fail for high titers. The response is erratic and non-predictive; thus, it mostly refrains from clinical utilization [16]. Bypassing agents are another modality that bridges coagulation by promoting a cascade through extrinsic pathways, especially in patients with a high-titer disease burden. Two bypassing agents have been approved for AHA, which are recombinant factor VIIA and plasma-derived anti-inhibitor coagulation complex (AICC or APCC). As per the EACH registry, both agents have equal efficacy and can be interchanged. The dose for recombinant VIIa is usually 70-90 mcg/kg and is repeated every two to three hours. For APCC/AICC, 50-100 U/kg dose is standard and repeated every eight to 12 hours with the maximum dosage of 200 U/kg in a day. These dosages can be titrated depending on the clinical picture, with the high dosage of 100 U/kg utilized for severe disease, leading to life-threatening illness. Treatment with both agents is continued until hemostasis is achieved with improvement in the clinical picture. Both recombinant VIIA and APCC/AICC have similar risk profiles, with thrombosis being the most critical complication. However, it should be noted that AHA can remit spontaneously, but fatal bleeds have been observed months after the initial diagnosis [2,15-17].

Immunosuppression at presentation should also be considered and is encouraged. EACH2 registry has the most extensive data reported on a cohort of 331 patients. It showed remission in 48% of patients on glucocorticoid monotherapy, 59% on a rituximab-based regimen, and 70% on a combination of glucocorticoid and cyclophosphamide [18]. The latest results from the Dutch cohort study by Schep et al. had almost the same experience with the aforementioned therapies and resulted in complete remission (CR) in 75% of the cases. Interestingly, it also showed that the most significant adverse effects in the treatment of AHA were infection, which occurred most frequently in steroid combination therapy than in the steroid monotherapy group. Mortality was linked mainly to infection. High-factor anti-FVIII titer, severe bleeding, and steroid monotherapy were associated with lower CR [19]. In pregnancy, steroid monotherapy decreases bleeding risk, and as far as the choice of immunosuppressive therapy, cyclophosphamide should be avoided. AHA associated with pregnancy can take a longer time to remit, but the spontaneous remission rate remains high. Relapses during subsequent pregnancies remain low but are not studied. It has been suggested that in nonbleeding hospitalized patients and those with prior aspirin or anticoagulation, thromboprophylaxis should be considered when factor VIII activity levels are above 50 U/dl [11].

## Conclusions

Pregnancy-associated acquired hemophilia A remains a mystery and has very scarce literature on its etiology, management in terms of bleeding, and hemostasis achievement. Given the abnormality in the intrinsic pathway, concerns must be raised, and AHA should be kept on top of differentials in pregnancy when aPTT is deranged. Delays in diagnosis, timely achievement of hemostasis, and prompt immunosuppression initiation in pregnancy-associated AHA can be disastrous.
